# A Gold Inlay

**Published:** 1907-10-15

**Authors:** Dayton D. Campbell


### A GOLD INLAY.

BY DAYTON D. CAMPBELL, D.D.S.

This inlay take the place of the large contour gold filling we are wont to put in the anterior teeth. It requires about half the time. The rubber dam need not be used, and, unlike the gold filling we spend hours to put in, cannot possibly be bitten out. There is no danger of pitting or peeling, and the inlay is susceptible of a much higher polish.

Prepare the tooth as you would for a large gold filling, by removing the pulp and filling the canal. Prepare the cavity without under cuts, leaving it saucer shaped, concave labio-lingually. This gives direct access to the root canal. With a plug-finishing bur and Arkansas stone leave the margins sharp. By drilling in a piece of wood find the diagonal of the square post to be used. Use a square post, because its four points of contact will assist you very materially in holding the base of the matrix while burnishing.

Now roughly burnish a piece of 40-gauge platinum plate to the floor and wall of the cavity (if it may be said to have walls), insert the pin, remove and tack with pure gold. Replace and burnish again to get the outline of the margins. Cut away the bulk of the surplus, burnish again and if the platinum has a tendency to spring up at the cutting edge, flow pure gold over the inner surface, then with a suitable gold plugger mallet every portion of the base to contact. Cut off the platinum pin you have been using to handle the work with and trim away the surplus plate flush with the margins.

On this base make and contour the gold filling, so to speak, out of wax or Parrs flux exactly as you want it to be when finished. Keep the wax flush with the margins of the tooth. Now cut a piece of inlay platinum foil large enough to wrap around the wax, smoothly covering the labial surface: bring the foil down over the cutting edge. Get the mesial or distal contour and leave the remaining lingual surface open. With a hot instrument touch the foil gently. This will cause it to stick so that it may be handled. Remove and paint the pin and platinum plate with whiting or rouge to prevent the gold from flowing where it is not wanted, and invest. Pick, melt or wash out the wax and solder with pure gold.

Old gold fillings may be utilized but should be melted into globules first. The labial surface may be pure gold and the remainder any karat your scraps afford. Again, the cutting edge can be made 22-karat and the remainder pure gold. Almost any degree of hardness may be obtained. After soldering, cool in water and cement to place. When the cement is sufficiently hard, finish as you would a gold filling. The platinum foil is removed with the first strip.

One would naturally think that the platinum plate along the margin would show, but it does not, owing to its becoming so thoroughly impregnated with the gold under the blow pipe.—*Dental Brief.*

